# Supplementary material for: Comparative multiomics analysis of cell physiological state after culture in a basket bioreactor
Source: Sci Rep. 2022 Nov 23;12:20161. doi: 10.1038/s41598-022-24687-4 (PMC9686226; doi:10.1038/s41598-022-24687-4)
Supplement: Supplementary file 1 — Supplementary Information 1. [file 41598_2022_24687_MOESM1_ESM.zip › raw data/Proteomic raw data/5.GOenrichment/EG.vs.CG/DOWN/EG.vs.CG.GO_Enrich.barplot.pdf]

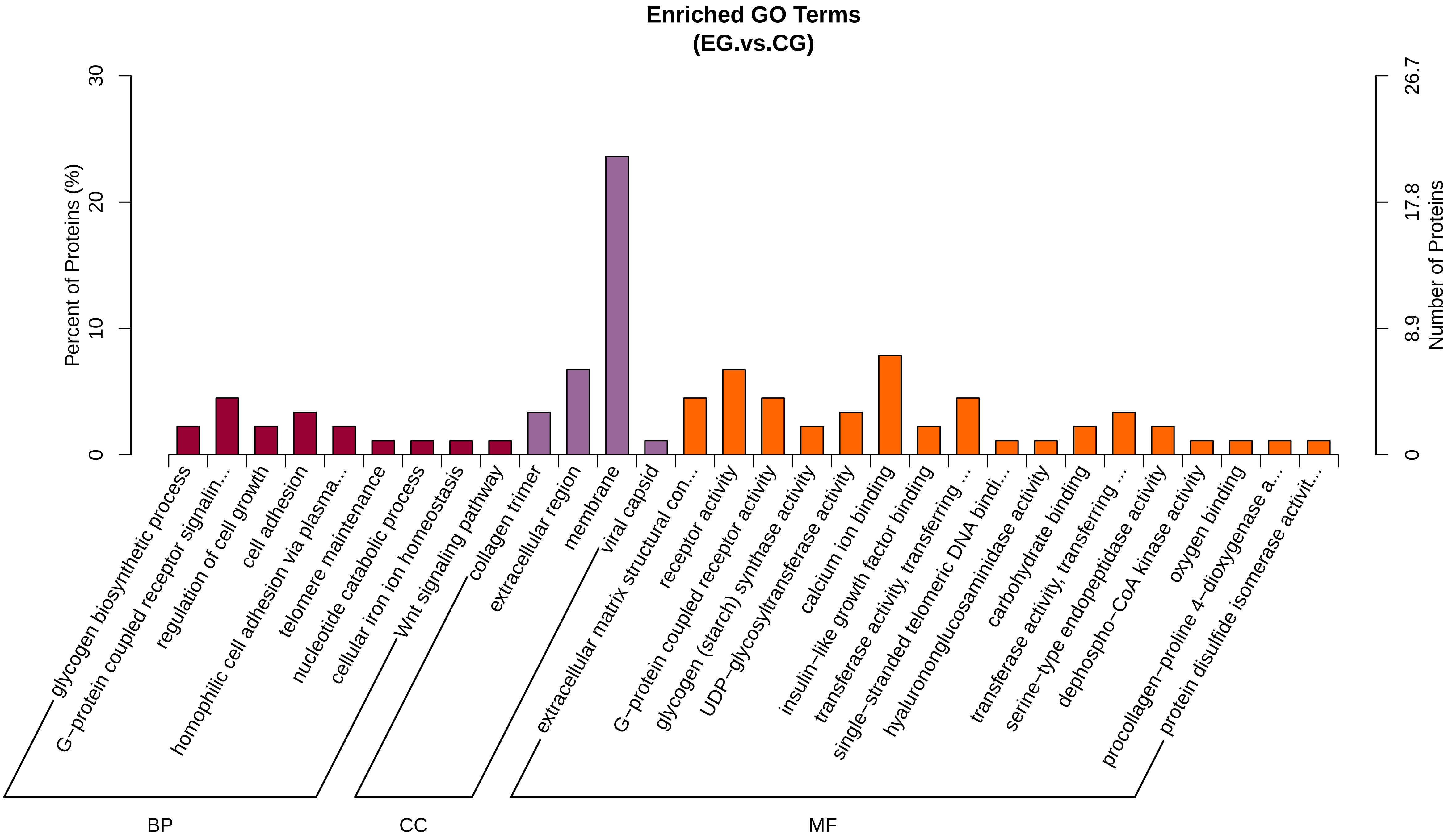

Enriched GO Terms
(EG.vs.CG)
Percent of Proteins (%)
Number of Proteins
0
10
20
30
0
8.9
17.8
26.7
glycogen biosynthetic process
G−protein coupled receptor signalin...
regulation of cell growth
cell adhesion
homophilic cell adhesion via plasma...
telomere maintenance
nucleotide catabolic process
cellular iron ion homeostasis
Wnt signaling pathway
collagen trimer
extracellular region
membrane
viral capsid
extracellular matrix structural con...
receptor activity
G−protein coupled receptor activity
glycogen (starch) synthase activity
UDP−glycosyltransferase activity
calcium ion binding
insulin−like growth factor binding
transferase activity, transferring ...
single−stranded telomeric DNA bindi...
hyaluronoglucosaminidase activity
carbohydrate binding
transferase activity, transferring ...
serine−type endopeptidase activity
dephospho−CoA kinase activity
oxygen binding
procollagen−proline 4−dioxygenase a...
protein disulfide isomerase activit...
BP
CC
MF
